# Clinicopathological factors associated with trichilemmal cysts

**DOI:** 10.1016/j.jpra.2025.11.013

**Published:** 2025-11-16

**Authors:** Emrah Işıktekin, Salih Kılıç, Bilgen Can, Betül Gözel

**Affiliations:** Department of Plastic Reconstructive and Aesthetic Surgery, Faculty of Medicine, Balıkesir University, Balikesir, Turkey

**Keywords:** Trichilemmal cyst, Proliferating trichilemmal tumor, Scalp tumor, Hair follicle neoplasms

## Abstract

Trichilemmal cysts (pilar cysts) are common adnexal tumors that mostly affect the scalp. They are usually benign, but can sometimes be malignant. Despite being common, there is limited data on what factors influence lesion size, multiplicity and risk of infection. The present retrospective observational study evaluated 27 patients who underwent excision of trichilemmal or related cystic tumous between January 2020 and June 2025 at a tertiary plastic surgery centre. The clinical nkvariables encompassed age, sex, diabetes mellitus (DM), hypertension (HT), tobacco use, Fitzpatrick skin type, and, in female patients, headscarf use and Ludwig hair loss score. Tumou size, multiplicity and infection were assessed. The mean age was 44.4 ± 15.6 years (range 17–82), with 59 % (16/27) females. The median tumor size was recorded as 21 mm [8,30] and the median duration from first awareness to excision was 18 months [8,27]. Infection was identified histopathologically in a minority of cases and showed trends toward association with DM and HT. Patients with multiple lesions were older (median 54 vs. 38.5 years, p=0.009) and had larger tumors (median 30 vs. 9 mm, p<0.0001). Linear regression revealed that age and DM independently predicted larger lesions, while HT was negatively associated. No correlations were identified with tobacco use, Fitzpatrick skin type, or headscarf status. These results emphasise the clinical importance of age and existing health conditions, especially diabetes, in affecting trichilemmal cyst development and the risk of infection. They provide new evidence for risk assessment, surgical planning and patient information.

## Introduction

Trichilemmal cysts (pilar cysts) and their variants, including proliferating and calcified forms, represent benign adnexal tumors most commonly located on the scalp. Although typically indolent, they may present as large or multiple lesions, raising cosmetic and clinical concerns. Nevertheless, the proliferating form has been shown to occasionally demonstrate malignant and even metastatic potential, as highlighted in a recent systematic review,^1^ underscoring the clinical importance of careful characterization and follow-up. Excision is the standard treatment. However, little is known about the influence of demographic, metabolic, and lifestyle factors on postoperative outcomes, lesion multiplicity, or tumor size. The present study aimed to evaluate these associations in a cohort of patients undergoing excision of trichilemmal and related cystic tumors.

Pilar cysts are common, affecting 5–10 % of the population.^2^ They are the most common cutaneous cyst on the scalp and the second most common cyst in the head and neck region.^3^ Pilar cysts are almost always benign, with malignant transformation occurring rarely. In 2 % of cases, single or multiple foci of proliferating cells lead to tumors, which are often called proliferating trichilemmal cysts.^4^ These grow rapidly and may also arise de novo. Although they are biologically benign, they can be locally aggressive, growing large and ulcerating. Rarely, malignant transformation leads to distant metastasis.^5^^,^^6^

Complete surgical excision with clear margins is the recommended treatment for all trichilemmal cyst variants; this approach is usually curative for benign and proliferating lesions and is critical for preventing local recurrence or malignant progression in atypical cases.^7^ Long-term follow-up is advised, especially for proliferating lesions, given the small risk of recurrence or malignant change.^7^^,^^8^

Recent genetic studies have revealed that hereditary trichilemmal cysts are caused by a two-hit, monoallelic mechanism involving the PLCD1 gene, which encodes phospholipase C delta 1. Both studies demonstrated that affected individuals carry a germline high-risk haplotype (most commonly p.S460L or p.Pro301Pro) and that a second somatic mutation (typically p.S745L) occurs on the same allele (in cis), triggering cyst development. This pattern deviates from Knudson's classical two-hit model, as both mutations affect a single allele rather than both copies of the gene. Functional assays confirmed that the cyst-specific PLCD1 variants lead to reduced enzymatic activity, thus supporting their pathogenic role. These findings provide strong evidence that trichilemmal cysts are genetically driven entities arising from a defined somatic-germline interaction in PLCD1, rather than merely sporadic benign lesions Research has demonstrated the existence of studies that provide substantial evidence for the proposition that trichilemmal cysts, which predominantly manifest as sporadic benign lesions, are not merely sporadic benign lesions, but rather genetically determined formations arising from a somatic-germline interaction defined in PLCD1.^9^^,^^10^ Literature on the subject also includes case reports on the development of trichilemmal cysts after trauma.^11^ Furthermore, the etiological links between trichilemmal cysts and infection, tobacco use, headscarf use, diabetes mellitus (DM), and hypertension (HT) remain to be elucidated. Previous literature on this subject has been largely confined to case reports and small series. The present study aimed to evaluate these associations in a cohort of patients undergoing excision of trichilemmal and related cystic tumors.

Although trichilemmal cysts are prevalent, the etiologic links with infection, multiplicity, DM and HT remain to be elucidated. Previous literature on this subject has been largely confined to case reports and small series. In contrast, our retrospective cohort study revealed statistically significant associations between DM, HT, and infection with cyst characteristics.

## Materials and methods

### Study design

A retrospective observational study was conducted at the Department of Plastic, Reconstructive, and Aesthetic Surgery, a tertiary health care centre. Patients who underwent excision of trichilemmal cystic tumors between January 2020 and June 2025 were subjected to a comprehensive evaluation. A comprehensive dataset encompassing patients' demographic information, comorbidities, Fitzpatrick skin types, tobacco use, lesion size, location, and time elapsed since initial detection was analyzed. Furthermore, the evaluation incorporated histopathological evidence of cyst infection, as documented in pathology reports, as well as headscarf use (defined as habitual scalp covering ≥12 h/day), and Ludwig hair score (I–III; grading system for female scalp hair thinning) among female patients.

Patients who did not wish to participate in the study, patients with incomplete data, and patients without outpatient clinic follow-up were excluded from the study. The patients included in the study had tricholemmal cysts or pilar cysts and proliferative tricholemmal cysts.

The study was granted ethical approval by the institutional review board (2025/11-5). All patients included in the study provided written consent for the use and publication of their data.

### Data collection and analysis

The following variables were included in the study: age, sex, DM, HT, tobacco use, headscarf use (females), Ludwig hair score (females), Fitzpatrick skin type, pathology group, anatomical region and time from detection to excision. The statistical analysis was conducted with the objective of investigating the correlation between postoperative infection, the presence of multiple tumors, and conditions believed to influence tumor size. Postoperative infection was defined according to CDC criteria within 30 days following surgery, as the presence of purulent drainage and/or a positive culture, or a surgeon's diagnosis of infection requiring antibiotic therapy. Follow-up data were obtained from routine clinic visits and, when applicable, telephone contacts documented in the medical record. Multiplicity was defined as the presence of ≥2 trichilemmal lesions documented in the same patient during the study period, irrespective of anatomical location or whether excised in the same or different sessions. Tumor size was abstracted from gross pathology reports as the largest diameter in millimeters. In instances where patients presented with multiple lesions, the index lesion was defined as the largest lesion and utilized for size-based analyses.

### Data sources and management

The data were derived from a comprehensive range of sources, including electronic medical records, operative notes, anesthesia reports and pathology archives, using a standardized form. When clinical and pathological measurements were available, the latter were given greater weight in order to minimize measurement variability. Two investigators reviewed a random 10 % sample of cases independently and any discrepancies were resolved by consensus. Missing values in secondary variables were deleted listwise. Prior to analysis, the data were de-identified.

### Statistical analysis

All statistical analyses were conducted using IBM SPSS Statistics version 29. Continuous variables are reported as mean ± standard deviation (SD) or median [interquartile range, IQR], and categorical variables as counts and percentages. Between-group comparisons employed the Mann-Whitney U test for continuous variables and Fisher's exact or Chi-square test for categorical variables, as appropriate. In light of the rarity of the postoperative infection outcome, a modelling approach involving Firth-penalized logistic regression was employed to mitigate the impact of small sample sizes and reduce bias. The results obtained are expressed as odds ratios (OR) with 95 % confidence intervals (CI). Multiplicity (solitary vs. multiple lesions) was examined using multivariable logistic regression, with adjustments made for age, sex, DM, HT, tobacco use, and time from detection to excision. In order to achieve normality, the tumor size was transformed using log-transformation, and subsequently modelled through the implementation of linear regression. The coefficients are presented as β (95 % CI), and when back-transformed, expressed as a percent change in tumor size. The model diagnostics encompassed residual and influence assessment, linearity checks for continuous predictors, and evaluation of multicollinearity (variance inflation factor <2). Robustness was assessed through the execution of sensitivity analyses, with the exclusion of giant lesions (defined as those exceeding 100 mm) and the restriction of analyses to scalp lesions only. The statistical significance was set at two-sided p<0.05.

## Results

A total of 27 patients were included. The median age of the patients was 41 [35,54], with 59 % (16/27) of the patients being female. The median tumor size was recorded as 21 mm [8,30]. The median time from first awareness of the lesion to surgical excision was 18 months [8,27], with a wide range from 4 to 110 months. reflecting frequent delays in presentation. The evaluation incorporated histopathological evidence of cyst infection occurring in a minority of cases. The application of a logistic regression model revealed no statistically significant predictors; however, trends suggested that DM (OR≈ 4.05), HT (OR≈ 6.50), and larger tumor size (OR≈ 14.5) were associated with higher infection risk. Multiple lesions were associated with older age (median 54 vs. 38.5 years, p=0.009) and larger size (median 30 vs. 9 mm, p<0.0001). With respect to comorbidities, 33.3 % (9/27) of participants had DM and 25.9 % (7/27) had HT, while 44.4 % (12/27) of participants reported frequent tobacco use. Due to the limited sample size and the small number of infection events, the logistic regression models demonstrated substantial statistical instability. Consequently, the odds ratios are reported as trend estimates without reliable confidence intervals or p-values.

Linear regression demonstrated that age (β=0.32, p=0.006, 95 % CI: 0.10–0.54) and DM (β=0.46, p=0.035, 95 % CI: 0.05–0.88) independently predicted larger lesions, while HT was negatively associated (β=−0.65, p=0.021, 95 % CI: −1.15 to −0.14). The final model yielded an adjusted R² of 0.31. Regression assumptions were verified and met. An overview of the cohort's dermatology-related history and risk factors is provided in Table 1. The majority of participants (70.4 %) (19/27) had never visited a dermatologist prior to diagnosis, and only 18.5 % (5/27) were aware of their cysts before presentation. indicating that these lesions are frequently incidental findings. The practice of regular self-examination was reported by only 22.2 % (6/27) of patients. This is indicative of limited awareness and surveillance. Among female patients, 43.8 % (12/27) reported regular headscarf use (at least 12 h a day), and nearly one-third had a Ludwig-Savin score of II or higher, reflecting clinically significant hair loss. The Fitzpatrick skin types demonstrated a balanced distribution, with 48.1 % (13/27) categorized as I – II and 51.9 % (14/27) classified as III – IV.

**Table 1:** Dermatology history, knowledge, and trichilemmal cyst risk factors of patients(n=27).

Variable	Category	n ( %)
Dermatology history	Previous dermatology visit	8 (29.6)
	No previous dermatology visit	19 (70.4)
Knowledge & surveillance	Aware of cyst before diagnosis	5 (18.5)
	Not aware / incidental finding	22 (81.5)
	Regular skin self-exam	6 (22.2)
	No regular surveillance	21 (77.8)
Risk factors	Diabetes mellitus (DM)	9 (33.3)
	Hypertension (HT)	7 (25.9)
	Tobacco use	12 (44.4)
	Headscarf use (females only, n=16)	7 (43.8)
	Fitzpatrick skin type I–II	13 (48.1)
	Fitzpatrick skin type III–IV	14 (51.9)
	Ludwig score ≥ II (females)	5 (31.3 of females)

### Case presentations

In addition to the overall cohort analysis, three cases are presented to highlight the clinical spectrum and surgical management of the largest and most challenging lesions observed in our series.

### Case 1

**Figure 1:**
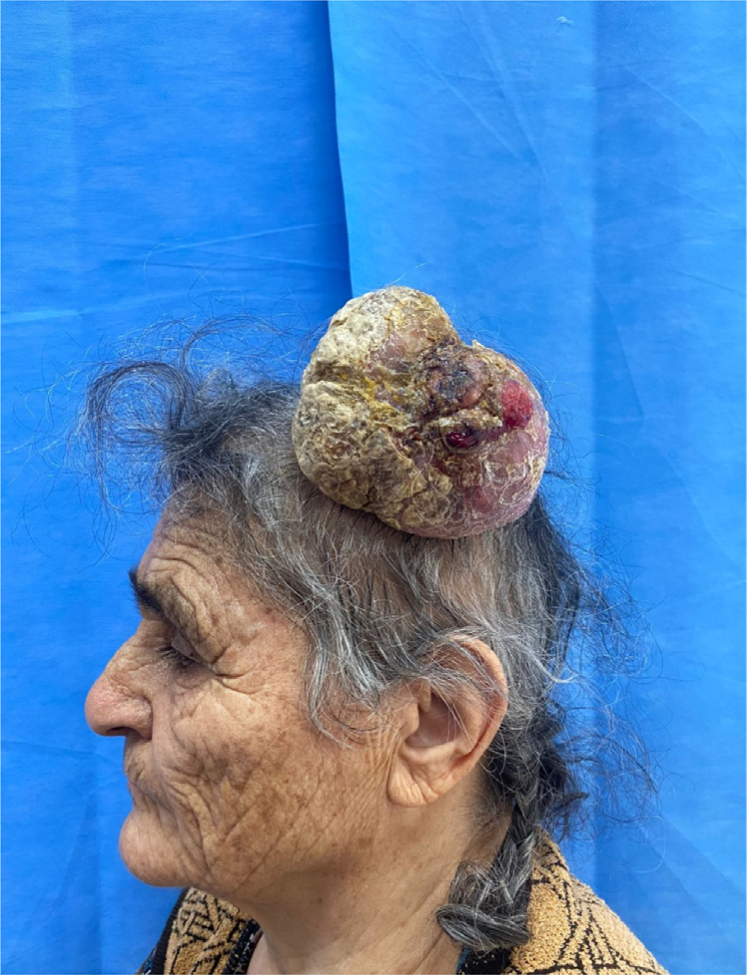
Large vertex scalp tumor in Case 1 demonstrating a well-circumscribed proliferating trichilemmal mass with intact overlying skin.

A 74-year-old woman presented with a huge, painless mass on the vertex of the scalp that had been slowly enlarging for about 10 years (Figure 1). The lesion measured 10 × 8 × 7 cm, with intact overlying skin and no fixation to the underlying bone. Imaging confirmed a well-defined heterogeneous tumor with calcifications but no intracranial extension. Complete excision was performed under general anesthesia, and the resultant defect (∼10 cm) was covered with a full-thickness inguinal skin graft. Histopathology confirmed a proliferating trichilemmal tumor without malignant features. The postoperative course was uneventful, and the patient was satisfied with the cosmetic outcome.

### Case 2

**Figure 2:**
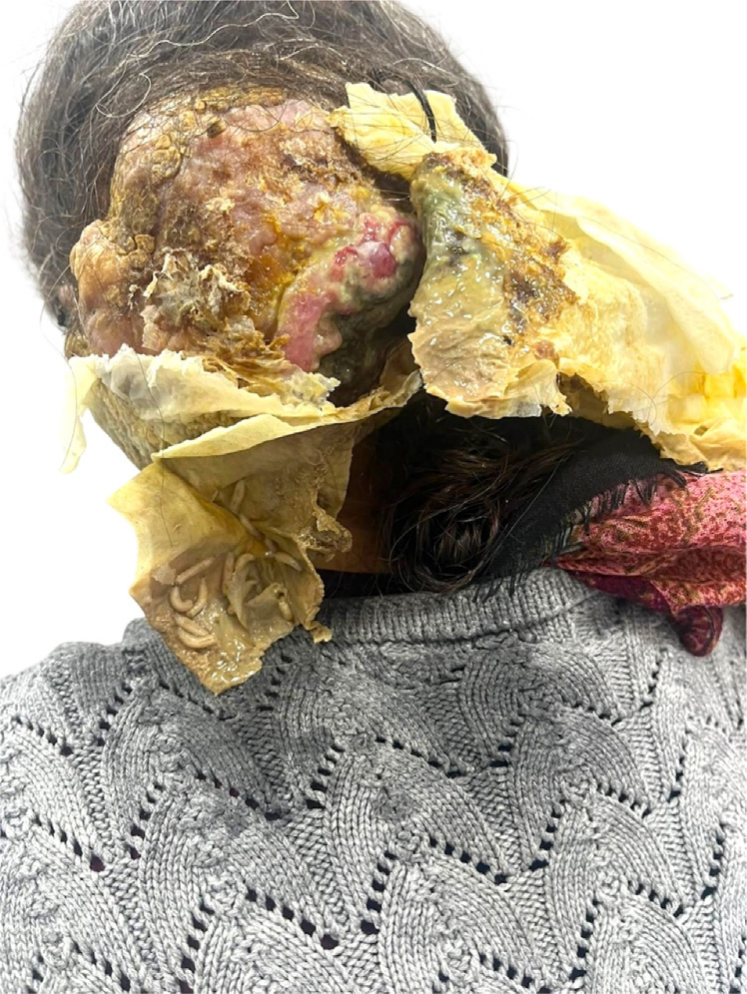
Neglected temporo-occipital scalp tumor in Case 2 showing ulceration, chronic discharge, and secondary myiasis.

**Figure 3:**
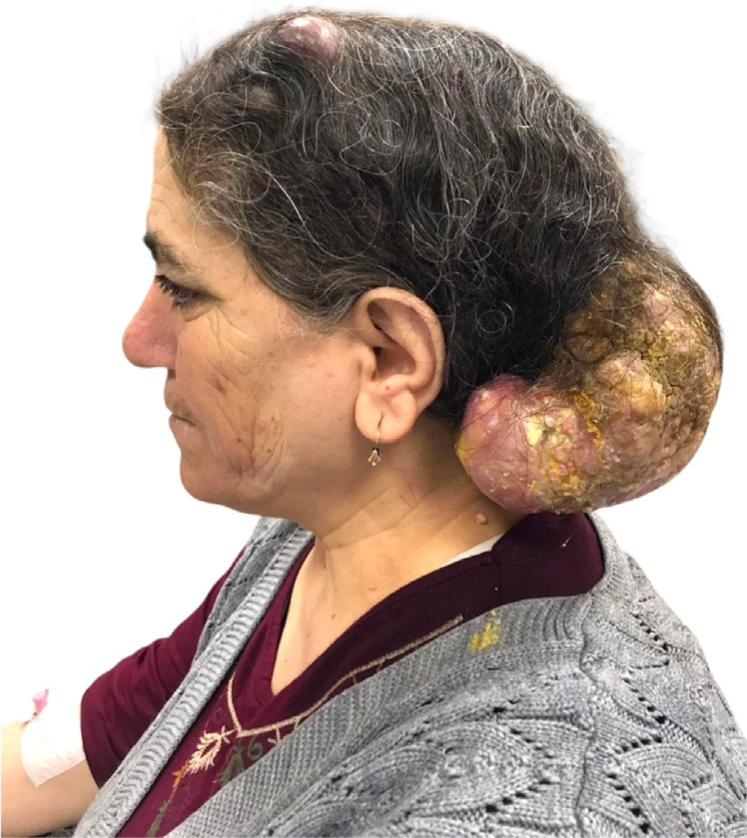
Lateral view of the giant temporo-occipital scalp tumor in Case 2 highlighting its external morphology, ulcerated surface, and overall lesion extent.

A 53-year-old woman presented with a neglected, malodorous tumor of the left temporo-occipital scalp, measuring 14 × 12 × 10 cm (Figure 2, Figure 3). The lesion had been present for 10 years, with recent ulceration, chronic discharge, and secondary myiasis. Intraoperatively, the mass was dissected free from the pericranium and reconstructed using a 15 × 12 cm full-thickness groin graft. Histopathology confirmed a proliferating trichilemmal cyst/tumor with extensive secondary infection but no malignant change. The wound healed well, and the patient remained disease-free at follow-up.

### Case 3

**Figure 4:**
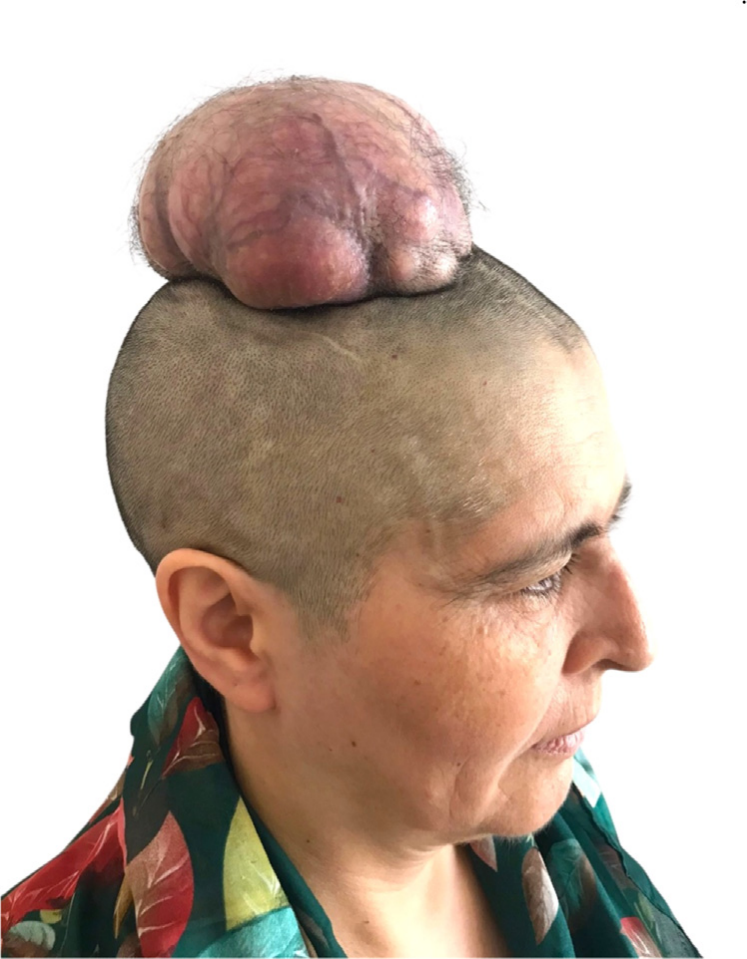
Parietal scalp tumor in Case 3 with five-year progressive growth, focal ulceration, and features consistent with a proliferating trichilemmal tumor.

A 57-year-old woman presented with a pedunculated parietal scalp mass measuring 13 × 6 × 6 cm. The lesion had grown progressively over 5 years, with partial surface ulceration but no evidence of bone involvement. Surgical excision was performed with a 6 cm graft reconstruction. Pathology revealed a benign proliferating trichilemmal tumor. At 1-year follow-up, the patient remained recurrence-free, with complete graft take and satisfactory cosmetic results (Figure 4).

## Discussion

Although the scalp remains the predominant location for trichilemmal cysts, the present study also revealed that lesions can manifest in atypical locations, including the lower extremities, face, trunk, and upper extremities. These findings emphasize the prevailing concept that trichilemmal cysts are conventionally regarded as scalp-limited adnexal tumors, yet their clinical spectrum is more extensive. This observation is corroborated by recent literature, including a case report of a pilar cyst arising on the dorsum of the hand—an area largely devoid of pilosebaceous units.^12^

The present findings underscore the clinical significance of the quantity of trichilemmal cyst lesions. In the patient series under consideration, patients with multiple lesions were found to be older and to have larger tumors. This finding suggests that the number of lesions may potentially reflect a broader clinical spectrum. This observation is of particular importance in light of recent reports describing malignant proliferative trichilemmal tumors (MPTTs) arising in patients with multiple trichilemmal cysts.^13^ Those with cysts should be evaluated if growing rapidly as this might indicate malignancy. Multiple cysts indicate a poor prognosis. The correlation observed between advanced age and increased lesion size is consistent with the clinical observations documented in the extant literature. The prevalence of trichilemmal tumors in elderly women is well documented, often manifesting as substantial scalp masses with a protracted growth history. For instance, a case of a giant occipital proliferating trichilemmal tumor in a 71-year-old female patient was treated with wide excision, emphasising the significance of adequate surgical margins and meticulous histopathological evaluation.^14^ In a similar vein, Davudov et al. documented a 69-year-old female patient afflicted with an occipital giant proliferating pilar tumor, necessitating intricate resection and flap reconstruction.^15^ Concurrently, Riaz et al. reported a rare instance of a giant proliferating trichilemmal tumor emerging behind the left shoulder, which was addressed with surgical excision and demonstrated no recurrence at the 6-month follow-up.^16^ Collectively, these cases demonstrate that, although most proliferating lesions remain histologically benign, their size, chronicity, and potential for malignant transformation necessitate heightened clinical vigilance, timely intervention, and comprehensive management. The present findings serve to corroborate these observations by demonstrating that advanced age is closely linked with greater tumor burden, thus supporting the clinical impression that both intrinsic factors and delayed presentation can culminate in challenging surgical scenarios.

In this series, no statistically significant correlation was observed between headscarf use among female patients and lesion size or number. This finding is consistent with a recent cross-sectional study conducted in Indonesia, which showed no significant difference in scalp moisture or pH between women who wore headscarves and those who did not.^17^ The results of this study are consistent with the hypothesis that the use of headscarves alone may not be a significant etiological factor in the development of trichilemmal cysts. Conversely, internal and systemic factors such as age and the presence of metabolic comorbidities are hypothesised to be more influential. However, further research is required to ascertain the extent to which environmental and cultural factors contribute to the pathogenesis of these lesions. The findings of this study can also be contextualized within broader clinical-pathological analyses of cutaneous cysts. In a 2-year retrospective study of 324 patients, trichilemmal cysts accounted for 8.9 % of cases and predominantly affected the scalp of middle-aged and elderly women.^18^ Complications such as rupture and inflammation were observed less frequently in trichilemmal cysts than in epidermal cysts.^18^ This research contributes to existing literature on the matter by demonstrating statistically significant associations between age, diabetes, and lesion size, and by establishing that histopathological evidence of infection in the present cohort is associated with both DM and HT. The study's findings indicate that systemic metabolic factors contribute to both tumor burden and secondary infection development.

The field of descriptive pathology is complemented by the analysis of molecular data, which is beginning to shed light on the biological basis of malignant progression. Recent analyses have confirmed alterations such as TP53 mutations and HER2 amplification in MPTTs, thereby supporting the concept that intrinsic tumor biology can interact with host factors to shape clinical behavior.^19^ Concurrently, smaller anatomic-clinical series have emphasized the role of proliferative trichilemmal cysts as intermediate forms between benign cysts and malignant variants, with ulceration, trauma, and chronic inflammation playing potential triggering roles.^20^ A plethora of studies have underscored the diagnostic challenges associated with differentiating proliferative trichilemmal cysts from squamous cell carcinoma. This has emphasized the significant role of immunohistochemistry and histological grading in treatment decisions.^21^ Molecular and genetic studies were not performed in the current study. However, the presence of existing findings suggests the importance of further research to elucidate the pathogenesis of the disease.

The rarity of cases further illustrates the malignant potential of these lesions. Squamous cell carcinoma has been reported to arise within the wall of a trichilemmal cyst, even in the absence of traditional risk factors,^22^ and collision tumors combining trichilemmal cysts with invasive carcinoma have also been described.^23^ Furthermore, there is documented evidence of progression from proliferating trichilemmal cyst to frank trichilemmal carcinoma, including cases of deep tissue invasion and aggressive clinical behavior, necessitating adjuvant radiotherapy.^24^ These observations emphasize the importance of considering malignant transformation or coexistence with carcinoma in the management of trichilemmal lesions, despite their rarity.

In the present cohort, no significant association was observed between Ludwig female pattern hair loss score or Fitzpatrick skin type and lesion size or multiplicity. This finding suggests that intrinsic hair density patterns and cutaneous phototype may play a limited role in the biological behavior of trichilemmal cysts. The Ludwig classification is a widely accepted framework for staging female pattern hair loss, and it provides valuable insight into androgenetic alopecia and scalp dermatology more broadly.^25^ In a similar vein, the Fitzpatrick scale, despite recent updates aimed at incorporating skin colour and ethnicity with greater precision, is predominantly utilized for the estimation of ultraviolet sensitivity and cosmetic outcomes, rather than adnexal tumor biology.^26^

This investigation underscores the clinical significance of age and metabolic comorbidities in patients with trichilemmal and related cystic tumors. While the occurrence of infection was infrequent, multiplicity exhibited a robust correlation with advanced age and increased tumor size. The correlation between diabetes and increased tumor size is consistent with the established role of metabolic dysregulation in cutaneous pathology. Contrary to predictions, hypertension demonstrated a negative correlation with tumor size. This may be indicative of a spurious finding due to the limited sample size.

No associations were identified with Fitzpatrick skin type, tobacco use, or headscarf status. These results suggest that environmental or lifestyle factors may play a lesser role than intrinsic or systemic factors.

To the best of our knowledge, no previous studies have systematically evaluated the impact of demographic and metabolic factors such as age, diabetes, and hypertension on trichilemmal cyst characteristics. The statistically significant associations observed in this cohort, particularly the link between age, diabetes, and lesion size, therefore represent novel findings in this domain. The results of this study provide unique data that contributes to the existing body of knowledge on adnexal tumors. The findings emphasize the significance of incorporating systemic comorbidities into the evaluation of patients with trichilemmal and related cystic tumors.

The study's retrospective design, coupled with the modest size of the cohort, leads to diminished statistical power and generalizability. The conduction of larger, multicentre studies is indicated to confirm these findings and to further elucidate the biological mechanisms underlying lesion growth and multiplicity.

## Conclusion

In patients diagnosed with trichilemmal and related cystic tumors, advanced age and the presence of diabetes mellitus have been observed to be associated with larger lesion size. Furthermore, multiplicity is more frequently observed in older individuals. The present findings underscore the pivotal role of demographic and metabolic factors in the development of tumor burden. The recognition of such associations may assist clinicians in risk stratification, optimising surgical planning, and providing postoperative counselling that is tailored to the individual patient.

## Patient consent

Written informed consent was obtained from all patients for the use of their clinical data and photographs in this publication.

## Funding

None.

## Ethical approval

This study was conducted in accordance with the Declaration of Helsinki. Ethical approval was obtained from the Ethics Committee of Balıkesir University Faculty of Medicine (2025/10–11).

## Declaration of competing interest

The authors declare that they have no conflicts of interest to disclose.
